# Clinical Course and Outcomes of COVID-19 Patients Admitted to the ICU of a Tertiary Care Hospital in Central India: A Cross-Sectional Study

**DOI:** 10.7759/cureus.54744

**Published:** 2024-02-23

**Authors:** Alka Modi Asati, Rakesh Patel, Kritika Singhal, Chakresh Jain, Sonali Tripathi

**Affiliations:** 1 Community Medicine, Shyam Shah Medical College, Rewa, IND; 2 Community and Family Medicine, All India Institute of Medical Sciences, Bhopal, Bhopal, IND; 3 Internal Medicine, Shyam Shah Medical College, Rewa, IND; 4 Women and Child Development, Kalpataru Gramodyog Samiti, Bhopal, IND

**Keywords:** covid-19, tertiary care, icu outcome, comorbidity, covid mortality

## Abstract

Background: Coronavirus disease 2019 (COVID-19) rapidly spread globally, leading to a pandemic significantly impacting individuals, communities, and economies worldwide. Public health measures such as social distancing, mask-wearing, and hand hygiene have been implemented globally to mitigate the spread of the virus. Many people recovered from COVID-19, but some cases needed intensive care unit (ICU) care, among whom most required mechanical ventilation (MV).

Materials and methods: This hospital-based cross-sectional study was done among 75 clinical or reverse transcriptase-polymerase chain reaction (RT-PCR) test-confirmed cases of COVID-19 infection admitted to the ICU of a tertiary care unit in India.

Results: A maximum number of patients, i.e. 47 (63%), were male, and 26 (35%) belonged to the age group of 41-60 years. The most common symptom was fever at the time of admission to the hospital. Comorbidity was reported in 21 (28%) patients. The majority of patients recorded a combination of hypertension and diabetes. The majority (n =34, 45%) of the patients stayed for ≤ 3 days in the ICU, and 46 (61%) deaths were recorded in the ICU during this period.

Conclusion: Delayed medical intervention, advanced age, male gender, and underlying health conditions like cardiovascular disease and diabetes can contribute to worse outcomes and increased mortality in COVID-19 patients.

## Introduction

The COVID-19 pandemic has affected millions of people globally, with many requiring hospitalization and intensive care unit (ICU) treatment. ICU treatment is necessary for patients with severe COVID-19 symptoms, including respiratory failure, pneumonia, and other complications. This infection started in China in December 2019 and became a pandemic caused by severe acute respiratory syndrome coronavirus 2 (SARS-CoV2) [1]. In India, the first case of COVID-19 was reported on January 30, 2020 [2]. India's first wave of the COVID-19 pandemic lasted from January to November 2020. While a few cases kept appearing, the second wave evolved from February to July 2021 [3].

Till May 2021, more than 164 million confirmed cases and more than 3.4 million deaths were attributed to COVID-19 [3]. Nearly 95% of people recovered from COVID-19, and around 3-5% of cases required ICU care [2], including mechanical ventilation (MV). The COVID-19 pandemic has affected many aspects of people's lives, including physical, social, emotional, and behavioral well-being. The country enforced different control measures like social distancing, partial and total lockdowns, closure of schools and businesses, and wearing of face masks to halt the spread of disease. Although such measures have helped flatten the epidemic curve, a COVID-19 resurgence was reported after resuming all public activities. This increased morbidity and mortality associated with COVID-19, especially during the second wave [2,4].

The COVID-19 vaccine development was an essential step towards controlling the pandemic. India's COVID vaccination drive was started on January 16, 2021, in a phased manner [5]. Even after all this, many people were seriously affected and were admitted to hospitals, especially during the second wave. Most of these patients were admitted to the ICU and would eventually require invasive MV because of diffuse lung injury and acute respiratory distress syndrome (ARDS). Most of the ICU reports from the United States have shown that severe COVID-19-associated ARDS (CARDS) is associated with prolonged MV and increased mortality [6]. There may be institutional and regional variations in ICU outcomes and mortality. The unsatisfactory ICU outcomes and high mortality observed during CARDS have raised worries about the use of MV. India, now with the largest population in the world, had difficulty in treating severe COVID-19 cases because the country had only 49,000 ventilators [6]. This disproportionate availability of resources led to different types of outcomes in COVID-19 patients; this study throws light on the clinical course of COVID-19 patients in the tertiary care units to determine clinical characteristics and outcomes of COVID-19 patients admitted to ICU. 

This article was previously posted to the medRxiv preprint server on June 23, 2023 (doi: https://doi.org/10.1101/2023.06.07.23290823).

## Materials and methods

This was a hospital-based cross-sectional study conducted in the Medicine ICU of Shyam Shah Medical College, Rewa, Madhya Pradesh, India, a tertiary care institute in central India, for one month in May 2021. The study was approved by the Institutional Ethical Committee of Shyam Sham Medical College (approval number: IEC/MC/2020-470 dated 08/01/2021). All RT-PCR-positive or clinically diagnosed (CT scan findings suggestive of COVID-19) patients admitted to the ICU and aged more than 12 years were included in the study. All pregnant patients, patients who could not respond with their attendants absent during data collection, and patients or attendants who did not give consent were excluded.

A convenient sampling technique was applied to select the participants and a total of 75 RT-PCR-positive or clinically diagnosed (CT scan findings suggestive of COVID-19) patients were approached in the final analysis.

Study tool and data collection

Patients fitting the inclusion criteria were approached in the medicine ICU, and consent was taken from patients or their attendants before the interview. The study tool was a structured proforma designed, validated, and used for data collection. Information was collected from various sources, including treatment sheets, patients, and their attendants. Data related to socio-demography, clinical characteristics, and laboratory parameters were collected.

Statistical analysis

Data were collected and compiled using Microsoft Excel (Microsoft Corporation, Redmond, Washington, United States). Continuous data were presented as median and interquartile range (IQR). Frequency and percentages were used for categorical variables. Wilcoxon rank sum exact test, Fisher’s exact test, and Pearson’s Chi-squared test are applied wherever necessary to know the association between variables. The value of p <0.05 was considered statistically significant. Analysis was done using R software (4.3.1 version; R Foundation for Statistical Computing, Vienna, Austria).

## Results

Out of 75 patients admitted to the ICU, the mean age was 53.66 (±17.19) years. Out of the total number of participants, 26 (35%) belonged to the 41-60 years age group, followed by 23 (31%) in the 21-40 years age group. Most of the study participants were residents of rural areas. Sociodemographic characteristics are presented in Table 1.

**Table 1:** Sociodemographic characteristics of patients (N=75)

Characteristic	Frequency (percentage)
Age (years)	
<20	6 (8.0%)
21-40	23 (31%)
41-60	26 (35%)
>60	20 (27%)
Sex	
Female	28 (37%)
Male	47 (63%)
Locality	
Rural	49 (65%)
Urban	26 (35%)

Table 2 shows that 51 (68%) patients were positive for COVID-19 infection. Only 48 (64%) patients gave a history of close contact with any COVID-19-positive individual. Although 24 (32%) of patients were COVID-19 negative, their clinical status was strongly suggestive of COVID-19 on CT scans. The most common symptom during admission to the hospital was fever in 24 (32%) patients, followed by breathlessness in 14 (18.6%) and cough in eight (10.7%). Twenty-one (28%) patients had some comorbidity. Out of these, four (19%) patients reported hypertension, which was the most common comorbidity, followed by diabetes in three (14.2%); hypertension and diabetes were the most common comorbidities found together reported in five (23.8%) patients. With regard to the duration of stay in the ICU, most of the patients, i.e. 34 (45%), were admitted for three days or fewer. A total of 21 (28%) required MV. Concerning outcomes, 29 (39%) were alive, and 46 (61%) patients did not survive at the end of the study period.

**Table 2:** Clinical profile and outcome of patients admitted to the ICU ^#^thyroid disorder, malignancy, morbid obesity, seizer disorder, severe anemia

Variables	Frequency (percentage)
COVID-19 test (N=75)	
Negative	24 (32%)
Positive	51 (68%)
Symptoms of patients at the time of admission in hospital (N=75)	
Fever	24 (32%)
Breathlessness	14 (18.6%)
Fever with chills	12 (16%)
Cough	08 (10.7%)
Generalized weakness	06 (08%)
Altered sensorium	04 (5.3%)
Loss of appetite	03 (04%)
Pain in abdomen	03 (04%)
Diarrhea	01 (1.3%)
Number of co-morbidities (N=75)	
0	54 (72%)
1	11 (15%)
2	9 (12%)
3	1 (1.3%)
Type of co-morbidity (N= 21)	
Diabetes	03 (14.2%)
Hypertension	04 (19%)
Diabetes + Hypertension	05 (23.8%)
Coronary artery disease	02 (9.5%)
Pulmonary disease	02 (9.5%)
Others^#^	05 (23.8%)
Outcome (N=75)	
Alive	29 (39%)
death	46 (61%)
Duration of stay in ICU (days) (4 (3.0, 8.0))^*^ (N=75)	
≤3	34 (45%)
4-7	21 (28%)
8-14	14 (19%)
>14	6 (8.0%)
Requirement of mechanical ventilation (N=75)	
Yes	21 (28%)
No	54 (72%)

Table 3 shows that the median hemoglobin was 12 gm/dl, and 45 (60%) patients had some form of anemia. The maximum number of patients, 59 (79%), had normal platelet count, 28 (37%) had normal C-reactive protein (CRP), and 67 (89%) reported D-dimer >0.5. The maximum number of patients, 46 (61.34%), had high-resolution CT score >15, and more than half of patients, 39 (52%), reported severe disease.

**Table 3:** Laboratory investigations of patients at the time of admission in ICU (N=75) ^#^Severe disease was defined as either of these: Respiratory rate >24/minute, oxygen saturation (SpO2) < 94% on room air, confusion, drowsiness, hypotension, sepsis, septic shock, or admission to ICU [7]. data given as n (%) and median (IQR), wherever indicated

Parameter	Values
Haemoglobin, median (IQR)	12 (10, 13.65)
Anaemia, n (%)	
No	30 (40%)
Mild	18 (24%)
Moderate	24 (32%)
Severe	3 (4.0%)
WBC, median (IQR)	12,000 (9,300, 15,800)
Platelet, median (IQR)	126,000 (112,000, 179,500)
Normal, n (%)	59 (79%)
Thrombocytopenia, (%)	16 (21%)
C-reactive protien, n (%)	
Negative	28 (37%)
Positive (raised)	47 (63%)
D-dimer, n (%)	
≤0.5	8 (11%)
>0.5	67 (89%)
HRCT score, median (IQR)	17.0 (14.5.0, 21.0)
<8 (Mild), n (%)	01 (1.3%)
8-15 (Moderate), n (%)	28 (37.34%)
>15 (Severe), n (%)	46 (61.34%)
Disease severity status^#^	
Mild to moderate, n (%)	36 (48%)
Severe, n (%)	39 (52%)

Table 4 shows the association of patients’ outcomes with their sociodemographic and clinical characteristics. Maximum patients from the age group > 60 years died, but this result was insignificant. Only the duration of stay in the ICU (p-value =0.001) and high-resolution CT score (p-value <0.001) showed significant association with outcome.

**Table 4:** Association of patient outcomes with sociodemographic and clinical characteristics ^#^WHO Criteria: normal, equal or above 12 gm/dl; Mild, 10-11.9; Moderate, 7-9.9; Severe, <7 data given as n (%) and median (IQR), wherever indicated

Characteristic	Alive, n (%)		Death, n (%)	p-value	
Age (years)				0.149	
<20		1 (3.4%)	5 (11%)		
21-40		9 (31%)	14 (30%)		
40-60		14 (48%)	12 (26%)		
>60		5 (17%)	15 (33%)		
Sex				0.933	
Female		11 (38%)	17 (37%)		
Male		18 (62%)	29 (63%)		
Address				0.974	
Rural		19 (66%)	30 (65%)		
Urban		10 (34%)	16 (35%)		
COVID-19 test				0.714	
Negative		10 (34%)	14 (30%)		
Positive		19 (66%)	32 (70%)		
Duration of stay in hospital (days)				0.001	
≤3		8 (28%)	26 (57%)		
4-7		16 (55%)	5 (11%)		
8-14		5 (17%)	9 (20%)		
>14		0 (0%)	6 (13%)		
Number of comorbidities					0.177
0		25 (86%)	29 (63%)		
1		2 (6.9%)	9 (20%)		
2		2 (6.9%)	7 (15%)		
3		0 (0%)	1 (2.2%)		
Heamoglobin, median (IQR)	11.20 (10.00,13.80)		12.00 (10.58, 13.00)	0.7	
Aneamia^#^				0.332	
No		12 (41%)	18 (39%)		
Mild		4 (14%)	14 (30%)		
Moderate		12 (41%)	12 (26%)		
Severe		1 (3.4%)	2 (4.3%)		
WBC, median (IQR)	13,000 (11,000, 13,300)		11,000 (8,300, 16,200)	0.036	
Platelet, median (IQR)	118,300 (118,000, 176,000)		140,000 (110,500, 180,000)	0.2	
Platelet				0.637	
Normal		22 (76%)	37 (80%)		
Thrombocytopenia		7 (24%)	9 (20%)		
C-reactive protein				0.286	
Negative		13 (45%)	15 (33%)		
Positive		16 (55%)	31 (67%)		
D-dimer				0.049	
≤0.5		6 (21%)	2 (4.3%)		
>0.5		23 (79%)	44 (96%)		
HRCT score	15 (13, 17)		20 (17, 22)	<0.001	
Mild (<8)	0 (0%)		1 (2.2%)		
Moderate (8-15)	20 (69%)		8 (17%)		
Severe (>15)	9 (31%)		37 (80%)		

## Discussion

Our study reported the sociodemographic characteristics, clinical profile, and outcomes of the 75 patients admitted to the ICU for COVID-19 infection in May 2021 in the tertiary care hospital. Compared with other studies, the mean age of study participants was almost similar, i.e., 53.66 years in our study and 59 and 64 years in the study of Sweden and Atlanta, respectively [8,9]. The current study reported that age and gender were not significantly associated with higher mortality. Similar results were reported in a study conducted in Saudi Arabia [10]. However, a study conducted in Sweden reported that age was significant and gender was not significantly associated with mortality [7]. Most of our study participants were male.

Around 68.8% of participants experienced at least one comorbidity in a study conducted by Assiri et al. [10], while in our study, only 28% of patients experienced some comorbidity. Hypertension was the most common comorbidity, followed by diabetes. Similarly, in a study conducted in Sweden, hypertension (39.6%) and diabetes (26.2%) were found to be the most prevalent comorbidity [8]. Auld et al. reported that hypertension (61.7%) was the most common, followed by diabetes (45.6%) [9]. The most prevalent symptom found was fever (32%), followed by breathlessness (18.6%) and cough (10.7%) at the time of admission to the hospital. In a study conducted in North India, cough (34.7%) was the most common symptom, followed by fever (17.4%) [7]. 

In the current study, 28% required MV, in contrast to 76% and 83.2%, respectively, in a study conducted by Auld et al. [8] and Oliveira et al. [11]. Inadequate resources, technical capacity, and early deaths might be the reason for this low proportion of patients requiring mechanical ventilation. A study from Sweden reported that 30.3% of patients died, and 31.3% were discharged [8]. Similarly, a study from Atlanta reported that 28.6% of patients died in the ICU, 67.7% were transferred out of the ICU, and 3.7% remained in the ICU [9]. A Central Florida study reported that out of 131 patients admitted to the ICU, 80.2% remained alive at the end of the study period, 70.9 % were discharged from the hospital, and 19.8% of patients expired [11]. In contrast, our study reported higher deaths in the ICU due to COVID-19, i.e., 61% died. Our study reported a higher mortality than other literature. The reason behind this high mortality may be a delay in the arrival of patients to the health facility, delay in treatment due to overcrowded health facilities, exhausted healthcare workers, and inadequate training. Further, the presentation of patients with severe infection or end-stage disease might also be a reason. Around the world, ICU mortality due to COVID-19 ranged from 20-62% [11]. Given the duration of stay in the ICU, a maximum (48%) was admitted for three days or fewer. The mean ICU stay duration was less in our study than in the studies conducted in Florida and Sweden [7,10]. This may be because the facility is a tertiary care institute, so patients with severe symptoms or end-stage disease were admitted in a higher proportion. 

Recommendations and limitations

It is needed to empower the health facility to accommodate a cohort of critically ill patients in one location. Appropriate training related to triage of patients, use of personal protective equipment, and development of standard treatment procedures are required for proper patient care.

The limitations of this study are that it was carried out only for one month during India's second wave of the COVID-19 epidemic, and the result cannot be generalized as the study was conducted in a Government hospital setting. 

## Conclusions

The present study analyzed the data of patients with COVID-19 admitted to ICUs in a tertiary care hospital, presenting baseline characteristics, laboratory parameters, and outcomes. The mortality was 61%. Elderly people >60 years, male gender, associated comorbidities, and >15 high-resolution CT scores were risk factors for mortality during the second wave of COVID-19. Preventive measures such as increased awareness regarding disease and comorbidities should be emphasized in populations with such risk factors, and enhancement of health facility infrastructure and services, readiness for disease outbreaks to mitigate the spread of COVID-19, as well as other emerging and re-emerging diseases and the resultant mortality, should be considered in priorities by the health authority.

## References

[1] (2020). The species Severe acute respiratory syndrome-related coronavirus: classifying 2019-nCoV and naming it SARS-CoV-2. Nat Microbiol.

[2] Andrews MA, Areekal B, Rajesh KR (2020). First confirmed case of COVID-19 infection in India: a case report. Indian J Med Res.

[3] (2022). COVID-19 coronavirus pandemic. https://www.worldometers.info/coronavirus/.

[4] (2022). Madhya Pradesh reports first Covid-19 positive cases. https://www.indiatoday.in/india/story/breaking-news-march-20-india-updates-coronavirus-madhya-pradesh-protest-china-italy-nirbhaya-hangings-1657672-2020-03-20.

[5] (2022). Covid-19 in India: Next phase of vaccine drive kick-starts today. https://www.hindustantimes.com/india-news/covid19-in-india-next-phase-of-vaccine-drive-kick-starts-today-101614552866500.html..

[6] Richardson S, Hirsch JS, Narasimhan M (2020). Presenting characteristics, comorbidities, and outcomes among 5700 patients hospitalized with COVID-19 in the New York City area. JAMA.

[7] Mohan A, Tiwari P, Bhatnagar S (2020). Clinico-demographic profile & hospital outcomes of COVID-19 patients admitted at a tertiary care centre in north India. Indian J Med Res.

[8] Larsson E, Brattström O, Agvald-Öhman C (2021). Characteristics and outcomes of patients with COVID-19 admitted to ICU in a tertiary hospital in Stockholm, Sweden. Acta Anaesthesiol Scand.

[9] Auld SC, Caridi-Scheible M, Blum JM (2020). ICU and ventilator mortality among critically ill adults with coronavirus disease 2019. Crit Care Med.

[10] Assiri A, Iqbal MJ, Mohammed A (2021). COVID-19 related treatment and outcomes among COVID-19 ICU patients: a retrospective cohort study. J Infect Public Health.

[11] Oliveira E, Parikh A, Lopez-Ruiz A (2021). ICU outcomes and survival in patients with severe COVID-19 in the largest health care system in central Florida. PLoS One.

